# Exploring the hemicellulolytic properties and safety of *Bacillus paralicheniformis* as stepping stone in the use of new fibrolytic beneficial microbes

**DOI:** 10.1038/s41598-023-49724-8

**Published:** 2023-12-20

**Authors:** Serigne Inssa Ngom, Soufiane Maski, Bahia Rached, Taha Chouati, Lydie Oliveira Correia, Catherine Juste, Thierry Meylheuc, Bernard Henrissat, Elmostafa El Fahime, Mohamed Amar, Christel Béra-Maillet

**Affiliations:** 1 Université Paris-Saclay, INRAE, AgroParisTech, Micalis Institute, 78350 Jouy-en-Josas, France; 2 Laboratoire de Microbiologie et Biologie Moléculaire, Centre National pour la Recherche Scientifique et Technique, Rabat, Morocco; 3 Département de Biologie, Faculté des Sciences, Université Mohammed V, Rabat, Morocco; 4 Collections Coordonnées Marocaines de Microorganismes, Centre National pour la Recherche Scientifique et Technique, Rabat, Morocco; 5 Plateforme Génomique Fonctionnelle, Unité d’Appui Technique à la Recherche Scientifique, Centre National pour la Recherche Scientifique et Technique, Rabat, Morocco; 6 Laboratoire de Chimie-Physique et Biotechnologies des Biomolécules et Matériaux/Equipe Microbiologie Biomolécules et Biotechnologies, Faculté des Sciences et Techniques, Mohammedia, Morocco; 7 Biologie médicale, Pathologie humaine et Expérimentale et Environnement, Faculté de Médecine et de pharmacie de Rabat, Rabat, Morocco; 8 Université Paris-Saclay, INRAE, AgroParisTech, Micalis Institute, PAPPSO, 78350 Jouy-en-Josas, France; 9 Université Paris-Saclay, INRAE, AgroParisTech, Micalis Institute, MIMA2, 78350 Jouy en Josas, France; 10 Architecture et Fonction des Macromolécules Biologiques, Centre National de la Recherche Scientifique, Aix-Marseille Université, 13288 Marseille, France; 11 Department of Biological Sciences, King Abdulaziz University, Jeddah, Saudi Arabia; 12 Department of Biotechnology and Biomedicine, Technical University of Denmark, Kgs. Lyngby, Denmark

**Keywords:** Microbiology, Dietary carbohydrates, Polysaccharides, Hydrolases, Carbohydrates, Enzyme mechanisms, Enzymes, Biochemistry, Biochemical assays, Whole genome amplification, Scanning electron microscopy, Analytical biochemistry, Bioinformatics, Genomic analysis, Mass spectrometry, Microbiology techniques, Microscopy, Proteomic analysis, DNA sequencing, Next-generation sequencing, Biological techniques, Sequencing, Sequence annotation, Microbiota

## Abstract

*Bacillus* strains from the Moroccan Coordinated Collections of Microorganisms (CCMM) were characterised and tested for fibrolytic function and safety properties that would be beneficial for maintaining intestinal homeostasis, and recommend beneficial microbes in the field of health promotion research. Forty strains were investigated for their fibrolytic activities towards complex purified polysaccharides and natural fibres representative of dietary fibres (DFs) entering the colon for digestion. We demonstrated hemicellulolytic activities for nine strains of *Bacillus aerius*, re-identified as *Bacillus paralicheniformis* and *Bacillus licheniformis*, using xylan, xyloglucan or lichenan as purified polysaccharides, and orange, apple and carrot natural fibres, with strain- and substrate-dependent production of glycoside hydrolases (GHs). Our combined methods, based on enzymatic assays, secretome, and genome analyses, highlighted the hemicellulolytic activities of *B. paralicheniformis* and the secretion of specific glycoside hydrolases, in particular xylanases, compared to *B. licheniformis*. Genomic features of these strains revealed a complete set of GH genes dedicated to the degradation of various polysaccharides from DFs, including cellulose, hemicellulose and pectin, which may confer on the strains the ability to digest a variety of DFs. Preliminary experiments on the safety and immunomodulatory properties of *B. paralicheniformis* fibrolytic strains were evaluated in light of applications as beneficial microbes' candidates for health improvement. *B. paralicheniformis* CCMM B969 was therefore proposed as a new fibrolytic beneficial microbe candidate.

## Introduction

The human gastrointestinal tract harbours trillions of microorganisms, commonly referred as “gut microbiota”. The gut microbiota maintains a symbiotic relationship with the host by performing various functions such as metabolizing undigested food components and influencing fundamental processes essential for health^1,2^. Among food components, dietary fibres (DFs) from fruits, vegetables, legumes, and cereals include resistant starch, and plant cell wall carbohydrates such as cellulose, hemicelluloses, and pectin that are not digested by host enzymes. These polysaccharides enter the colon where they serve as substrates for intestinal bacteria, and in particular for primary fibre degraders considered “keystone” species of a trophic network that benefits the host^3^. These bacteria initiate the digestion of a variety of complex polysaccharides combining different sugar monomers and glycosidic bonds, thanks to a myriad of enzymes with complementary activities, mainly from diverse glycoside hydrolase (GH) families^4^. Hemicelluloses include (1,4) and (1,3;1,4) β-D-linked glycans such as xylans, xyloglucan, lichenan, β-glucans and mannans which are degraded by GHs such as xylanases, xyloglucanases, lichenases, and endoglucanases with the help of oligo- and monosaccharidases until simple sugars. By entering the metabolic pathways of fermentative bacteria, simple sugars fuel the microbiota towards the production of Short-Chain Fatty Acids (SCFAs). As a main source of energy in the host, SCFAs play a central role in gastrointestinal epithelial cell integrity, glucose homeostasis, lipid metabolism, appetite regulation, and immune function^5^.

Some recent pieces of evidence suggest that deficiency in SCFA production and depletion of fibre-degrading bacterial members have been associated with gut inflammatory disorders and type 2 diabetes^6–8^. Thus, modulating the gut microbiota through fibre-degrading bacterial members could be a promising approach for restoring gut homeostasis. In this context, we aimed to characterise novel fibre-degrading bacteria as new beneficial microbe candidates with promising health benefits and safe properties. *Bacillus* strains were chosen on the basis on the genus long probiotic history (isolates, mix of strains, or fermented products). Probiotics are defined by the Word Health Organization (WHO) as “live micro-organisms which, when administered in adequate amounts, confer a health benefit on the host”^9^. Traditionally, probiotics mainly encompass lactic acid bacteria and bifidobacteria, with a wide range of beneficial effects, generally strain-dependent^10^. More recently, due to a better understanding of the microbiome, the landscape of probiotics candidates has evolved towards the concept of next-generation probiotics, and has highlighted beneficial commensals such as *Faecalibacterium prausnitzii* and *Akkermansia muciniphila*, with targeted health benefits and therapeutic potential in metabolic diseases^11^.

The present study aims to extend this concept of next-generation probiotics by screening *Bacillus* strains for their fibrolytic function, specifically targeting the complex polysaccharides that enter the colon during digestion. DFs metabolism is the main function of gut microbiota, assumed by fibrolytic and glycolytic bacteria, and the first step in a microbial trophic network crucial to host homeostasis. *Bacillus* species are known to metabolize a variety of complex dietary fibres^12^, increasing levels of SCFA-producing bacteria^13,14^, and to have anti-obesity and anti-diabetic properties^15^. Although this is still a small niche market for humans, *Bacillus* species like *Bacillus licheniformis* are already used as probiotics in animals and humans. However, their fibrolytic function have been poorly explored. Recently, we reported for the first time a strain of *Bacillus paralicheniformis*, isolated from Moroccan collections, which produces extracellular lichenase and xylanase activities suitable for biotechnological applications^12^. In the present study, forty strains of *Bacillus aerius*, some re-identified as *B. licheniformis* and *B. paralicheniformis*, were included for comparative enzyme assays, genomic analyses for CAZyme profiles, and preliminary safety assessment of a selection of fibrolytic strains, according to the European Food Safety Authority (EFSA) recommendations. Interactions with intestinal epithelial cells (IEC) were assessed for immuno-modulatory properties of fibrolytic *Bacillus* strains. These experiments identified *B. paralicheniformis* CCMM B969 as a fibrolytic beneficial microbe candidate for further experiments in preclinical models.

## Results

### Functional screening of the *B. aerius* CCMM strains revealed hemicellulolytic activities

Forty strains previously identified as *B. aerius*^16^ were selected from the CCMM to assess their capacities to hydrolyze and metabolize hemicellulose sources from purified polysaccharides. Functional screening of the 40 strains for lichenase and xylanase activities was performed using the basal salt solution (BSS) agar medium supplemented with lichenan and xylan, respectively, as the sole carbon source. Depending on the nature of the polysaccharides used, the growth of *Bacillus* strains was first tested on solid media. Nine strains: CCMM B774, CCMM B940, CCMM B951, CCMM B954, CCMM B960, CCMM B966, CCMM B969, CCMM B943, and CCMM B945 were able to grow on the mentioned substrates (not shown). Colonies were irregular or circular, with different sizes and either smooth or rough, as described for *Bacillus* strains^17^.

**Figure 1:**
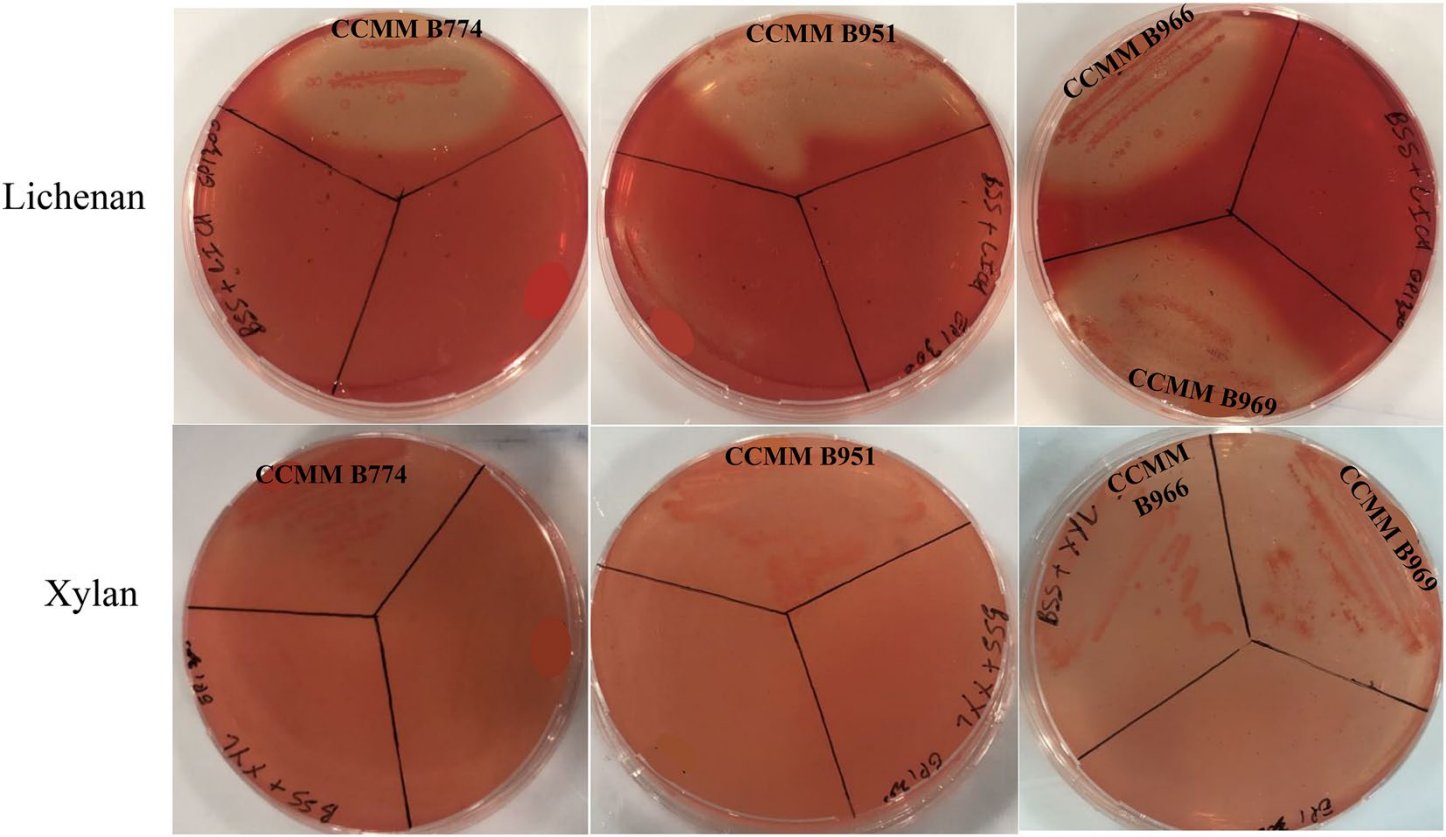
Detection of lichenase and xylanase activities of *Bacillus* CCMM strains grown in BSS agar medium supplemented with 0.5% (w/v) lichenan or xylan for 48 h at 37 °C. Congo red staining revealed clear halos around the streaks in the presence of enzymatic activities.

Strains growing on purified polysaccharides were then assessed for enzymatic activities. Clear halos around the streaks were observed for 9 of the 40 strains. Among them, five strains: CCMM B774, CCMM B951, CCMM B966, CCMM B969 (Fig. 1), and CCMM B940^12^, showed activities on both polysaccharides.

### Multilocus sequence typing confirmed the re-identification of *B. (para)licheniformis* hemicellulolytic strains

To assess and compare the phylogenetic classification of the nine hemicellulolytic *Bacillus* strains to other strains from the literature, we used different molecular methods based on single gene homology (16S rRNA and *gyrB* genes), multilocus sequence typing (MLST) scheme, or genome sequence comparison. The 16S rRNA gene sequences were all belonging to the genus *Bacillus*, with the highest similarity scores ranging from 99.8 to 100%. The closest species were *B. licheniformis* and *B. paralicheniformis*. A Neighbor-joining phylogenetic tree, based on 16S rRNA sequences of the isolates and type-strains retrieved from the GenBank database, separated our strains into two groups: strains CCMM B774, CCMM B969, CCMM B940, CCMM B951, and CCMM B943 were close to *B. paralicheniformis*, while the strains CCMM B960, CCMM B945, CCMM B966, and CCMM B954 were closely related to *B. licheniformis* (Fig. 2a).

**Figure:**
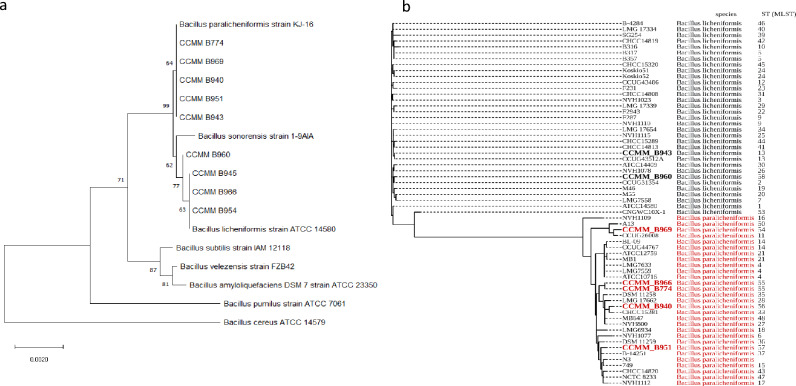
(**a**) Neighbor-joining phylogenetic tree based on 16S rRNA sequences of *Bacillus* isolates. The phylogenetic tree was inferred using the Neighbor-Joining method^62^. The percentage of replicate trees in which associated taxa clustered in the bootstrap test (1000 replicates) is shown next to the branches (bootstrap values ≥ 60). Evolutionary analyses were conducted in MEGA X^63^. The bar indicates 0.0020 substitution per site. rRNA gene sequences are presented with the corresponding species name and strain, or with the CCMM reference. (**b**) Multi Locus Sequence Typing (MLST) analysis of *B. licheniformis* and *B. paralicheniformis* strains. The phylogenetic tree was generated in iTOL v4 integrated into the PubMLST database, based on the internal fragments of six housekeeping genes. Seven *Bacillus* CCMM strains were submitted to the database. *Bacillus* isolates are presented with the corresponding species and strain names.

It has been previously described that gene *gyrB* could provide better species discrimination than 16S rRNA gene sequences in the *Bacillus subtilis* group^18^. For this reason, we used an intragenic fragment of *gyrB* to compare the strains using a new Neighbor-joining phylogenetic tree. The same clustering was obtained for *B. licheniformis* and *B. paralicheniformis* species, except for strains CCMM B966 and CCMM B943 (Fig. S1).

We then used the highly discriminant MLST method to compare the *Bacillus* strains to a more extensive set of previously published strains. Six housekeeping genes were used to discriminate between *B. licheniformis* and *B. paralicheniformis* (see “Methods” section; Table S1). Results showed that the identity of our loci ranged from 98 to 100% compared to the existing loci. A Neighbor-joining phylogenetic tree based on concatenated sequences distinctly separated all sequences into two different clades corresponding to *B. licheniformis* and *B. paralicheniformis* species (Fig. 2b). Hemicellulolytic strains CCMM B960, and CCMM B943 are classified as *B. licheniformis* while strains CCMM B969, CCMM B966, CCMM B774, CCMM B940, and CCMM B951 clustered with *B. paralicheniformis*. This result is coherent with the clustering based on the housekeeping gene *gyrB.* We, therefore, can conclude that the strains CCMM B945, CCMM B954, CCMM B960, and CCMM B943 should be reclassified as *B. licheniformis*, and the strains CCMM B969, CCMM B940, CCMM B774, CCMM B966, and CCMM B951 as *B. paralicheniformis* (Table S1). Furthermore, MLST data showed that all *B. paralicheniformis* strains have new sequence types (ST 54 to 57), as well as *B licheniformis* CCMM B960 (ST58), and assume unique positions in the intraspecies phylogenetic tree (Fig. 2b; Table S1).

The re-identification of the *Bacillus* strains is fully consistent with whole-genome sequencing and species belonging to *B. paralicheniformis*: CCMM B774, CCMM B951, CCMM B969, and CCMM B940, and to *B. licheniformis*: CCMM B943 (see the genomic features described below).

### *B. paralicheniformis* produced xylanase, and lichenase activities on dietary fibres, unlike *B licheniformis*

To assess the capacity of *Bacillus* CCMM strains to degrade natural plant fibres representative of DFs entering the colon for microbial digestion, we selected three different sources of the main fruits and vegetables consumed in Europe, namely apple, orange and carrot, with different expected ratio of cellulose, hemicelluloses, and pectin (Table S2).

**Figure 3:**
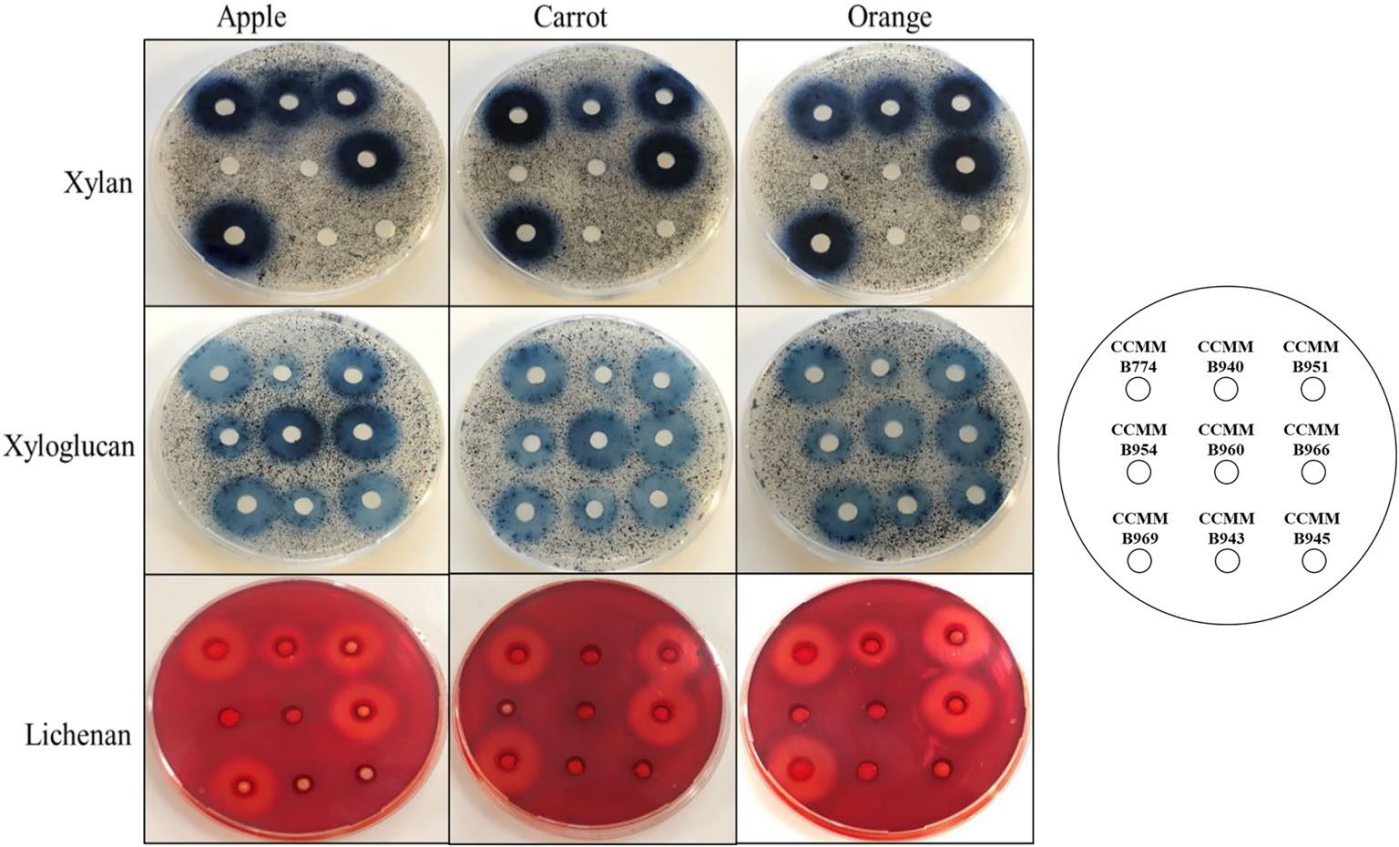
Detection of xylanase, xyloglucanase and lichenase activities of *Bacillus* CCMM strains, using agar-well plate assays with concentrated extracellular proteins from cultures in BSS medium and 0.5% natural substrates. Agar plate BSS medium was supplemented with 0.1% (w/v) lichenan, xylan or xyloglucan; Plates were incubated overnight at 37 °C. Clear and blue halos around the wells indicate a positive sample for the corresponding glycoside hydrolase activity.

The nine hemicellulolytic *Bacillus* strains were investigated for various GH activities using cultures in BSS medium supplemented with carrot, orange, and apple peels. All nine strains grew well in the presence of the natural fibres. Significant and reproducible enzymatic activities were detected in extracellular proteins using agar-well plates. Interestingly, all strains produced xyloglucanase activities (moderate activity for B940) (Fig. 3), whereas only the five strains of *B*. *paralicheniformis* (CCMM B774, CCMM B940, CCMM B951, CCMM B966, CCMM B969) displayed xylanases and lichenases.

The *B paralicheniformis* strains were then able to degrade efficiently all three complex polysaccharides from natural substrates. No significant differences were visible for polysaccharide degradation between cultures, except for the absence of lichenase activity in B940 when grown on the carrot.

**Figure 4:**
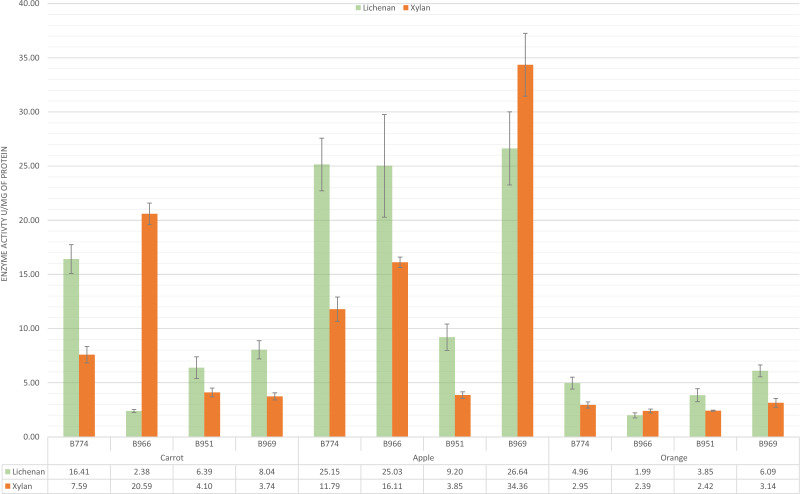
Enzymatic activity of extracellular proteins of *B. paralicheniformis* CCMM strains grown in BSS medium supplemented with carrot, apple and orange peels. Activity was measured by quantification of reducing sugars released after enzyme incubations with lichenan or xylan substrate. One international unit (IU) was defined as the amount of enzyme which produced 1 µmol of equivalent glucose or xylose in 1 min.

Lichenase and xylanase activities of the four most active strains (*B. paralicheniformis* CCMM B774, CCMM B951, CCMM B966, and CCMM B969) for all three polysaccharides, were then quantified (Fig. 4). GH activities were strain-dependent and varied according to the carbohydrate sources present in the culture medium. Lichenase and xylanase activities were highest for strains cultivated on apple peels, which could be related to the difference in glycan structure between DFs. *B. paralicheniformis* CCMM B969 produced the maximum lichenase activity with 26.64 ± 3.37 IU/mg of proteins and xylanase activity with 34.36 ± 2.90 IU/mg of proteins. The lichenase activity of strain CCMM B969 was 3 and 4 times lower when cultured in BSS carrot and BSS orange, respectively. Furthermore, the xylanase activity of B969 decreased 9 to 11 times on these substrates, compared to apple. *B. paralicheniformis* CCMM B966 had the highest xylanase activity in the BSS carrot (20.59 ± 1 IU/mg of proteins), which is ten times higher than in the BSS orange medium. Finally, the four *B. paralicheniformis* strains had higher activities than the strain CCMM B940 from our previous study^12^.

The carbohydrate fermentation patterns of these four hemicellulolytic strains showed that *B. paralicheniformis* used a wide variety of simple sugars, including d-glucose, d-fructose, d-lactose, sucrose, d-maltose, d-cellobiose, d-ribose, d-xylose, trehalose, L-arabinose, mannose, as well as sugar alcohols, glycosides, starch, and glycerol (Table S3), suggesting versatile metabolic abilities.

### *B. paralicheniformis* encodes multiple GH genes for efficient plant polysaccharide degradation

We compared the genomes of *B. paralicheniformis* CCMM B774, CCMM B940, CCMM B951, and CCMM B969 to that of *B. licheniformis* CCMM B943 for CAZyme annotation. The genome sequences of the four *B. paralicheniformis* strains encoded 128 CAZymes, including 67 GHs belonging to 32 GH families and sub-families, 13 Carbohydrate Esterases (CEs), 8 Polysaccharide Lyases (PLs), 1 Auxiliary Activity (AA), and 39 Glycoside Transferases (GTs). In comparison, only 59 GHs were encoded by *B. licheniformis* CCMM B943 (Table S4; Fig. 5).

**Figure:**
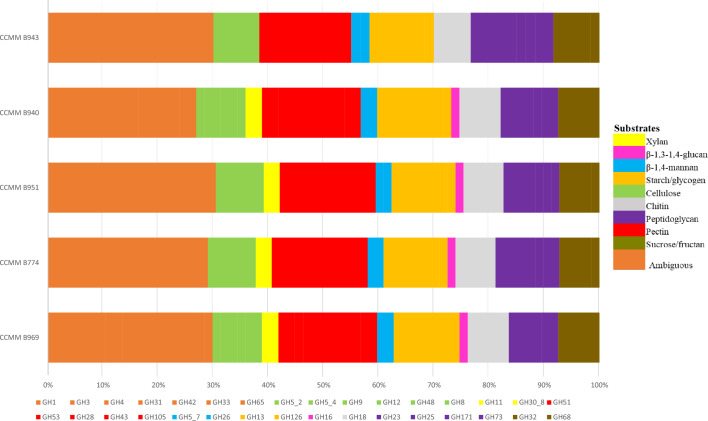
Overview of GH families and their predicted target substrates from *B. licheniformis* (CCMM B943) and *B. paralicheniformis* (CCMM B940, B951, B774, and B969) genomes. Assignment of ORFs to GH families was done according to the CAZy database (http://www.cazy.org/).

The GH families include predicted enzymes involved in the degradation of various complex carbohydrates found in DFs in humans. The GH5_2, GH5_4, GH9, GH12, and GH48 cellulases were observed in all strains (Fig. 5). However, the GH8 was not found in *B. licheniformis*. The pectinases, namely GH28, GH43, GH51, GH53, and GH105, were also present in both species and all strains. This is also the case for the mannanases, GH5_7 and GH26, involved in hemicellulose breakdown and for the fructanogenic GH32 and GH68 enzymes. Interestingly, GHs specifically dedicated to xylan degradation, namely GH11, and GH30_8, as well as the GH16 lichenase, were only found in *B. paralicheniformis*, consistent with the absence of xylanase and lichenase activities in *B. licheniformis* using enzymatic assays (Fig. 3). GH1, GH3, GH4, GH31, and GH42, present in all strains, may be involved in the metabolism of simple sugars, while GH13 and GH126 are probably related to starch metabolism. For all GH activities, predicted Carbohydrate-Binding Modules (CBM) were found in the genomes of all *B. (para)licheniformis* CCMM strains (Table S4), thus conferring GHs the ability to bind to various polysaccharides such as cellulose, xylan, lichenan, starch, and fructans, and to enhance fibre degradation. Finally, the hydrolytic enzymes GH23, GH25, GH73, and GH171 could reflect antimicrobial activities in *B. (para)licheniformis*.

### *B. paralicheniformis* secretome is mediated by the nature of plant carbohydrate sources

**Figure 6:**
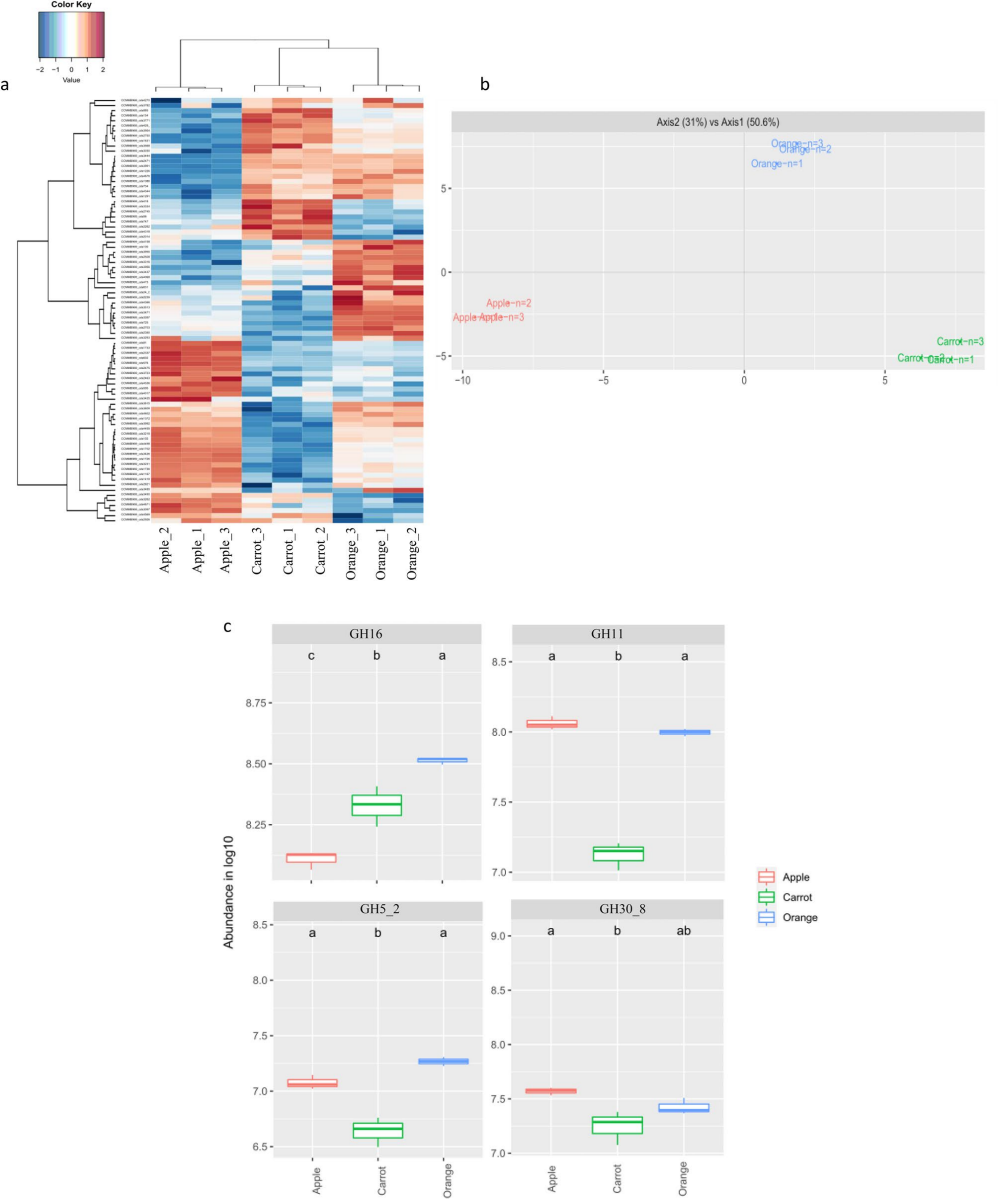
(**a**) Heat map comparison, (**b**) Principal Component Analysis of expression patterns, and (**c**) Abundance of selected CAZymes detected in the secretomes of *B. paralicheniformis* CCMM969 during growth on different carbon sources. Groups with different letters exhibit significantly different growth rates at a 95% confidence interval according to a Kruskhal–Wallis rank sum test, followed by a Dunn posthoc pairwise comparison test.

The secretome of *B. paralicheniformis* CCMM B969 was examined from bacteria grown on natural apple, carrot, and orange fibres, and analyzed by LC–MS/MS (Fig. 6). We identified 84 extracellular proteins as significantly abundant, and the secretome profile was substrate-dependent (Fig. 6a, b). Nineteen CAZymes were produced in all conditions including 14 GHs, 3 PLs, 1 AA, and a carbohydrate-binding module. GH11 and GH30_8 that degrade xylans were more abundant in the culture supplemented with apple (Fig. 6c), which is coherent with the highest xylanase-specific activity we noticed with this substrate. GH5_2 and GH16, rather involved in cellulose and lichenan degradation, respectively, were more abundant with orange. All four enzymes have a signal peptide for secretion. Natural fibres induced secretion of these different CAZymes according to their GH family and substrate specificity.

### *B. paralicheniformis* produced extracellular decorated filaments resembling exopolysaccharides when cultivated on natural carbohydrates

**Figure 7:**
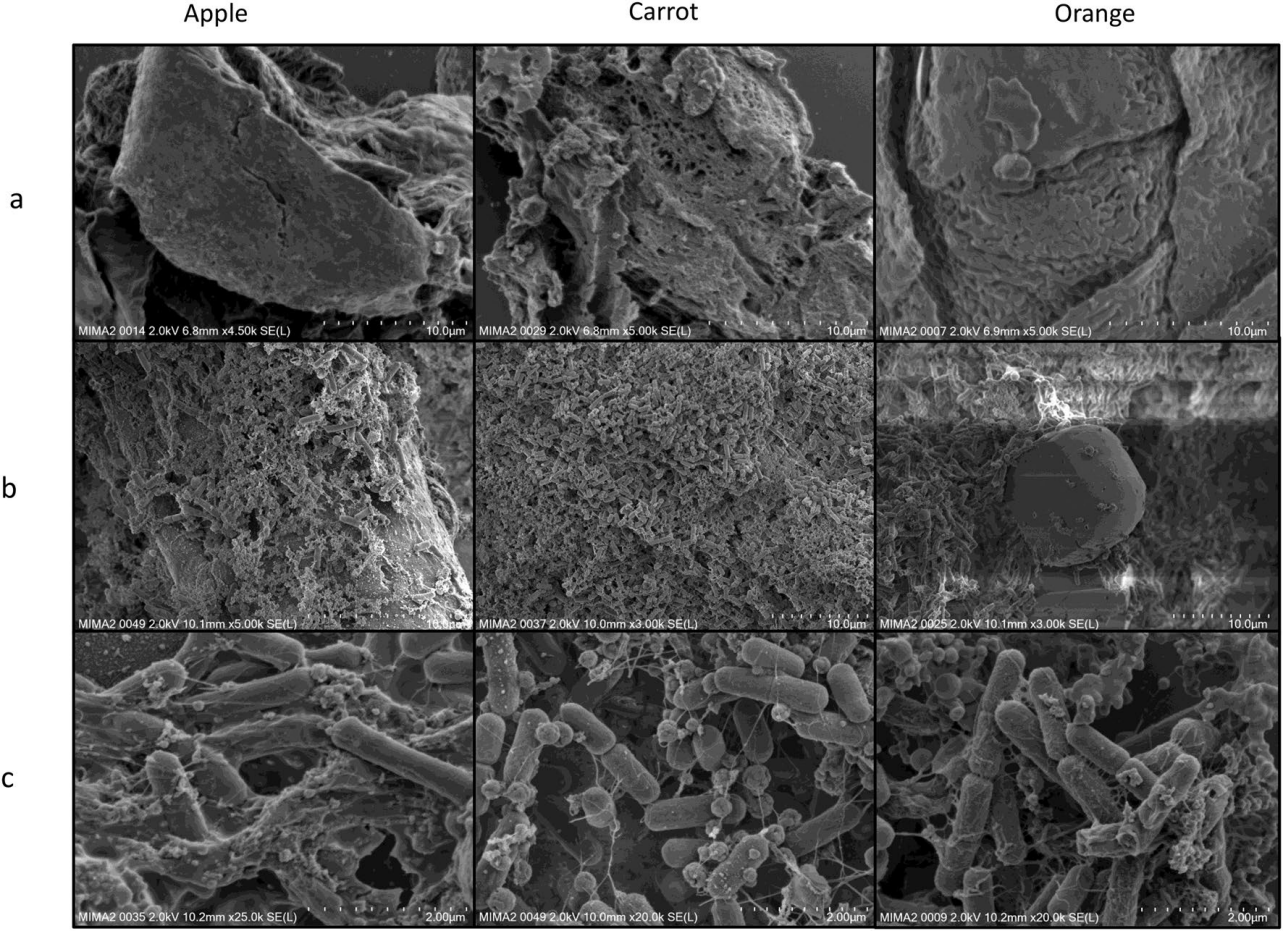
(**a**) Scanning electron microscopy (FEG-SEM) of natural fibres without bacteria, and (**b**,** c**) after incubation with *B. paralicheniformis* CCMM B951 for 48 h at 37 °C. Observations were made at different magnifications. Photos @INRAE—Thierry Meylheuc, ISC MIMA2, MICALIS, INRAE Jouy-en-Josas, France.

SEM analysis revealed the interactions of hemicellulolytic *Bacillus paralicheniformis* with natural fibres in vitro, using cultures in BSS medium supplemented with apple, carrot, or orange peel fibres. Before inoculation, fibres displayed a smooth and rigid surface (Fig. 7a). The rod-shaped bacteria then colonised the surface of the three fibres (Fig. 7b). Examination of the colonised cell surfaces at higher magnification showed that the bacteria were trapped in filamentous structures of an organised network with regular aggregates, for all three natural fibres (Fig. 7c), which is similar to extracellular polymeric substances (EPS) found in *B. subtilis*^19^, and mainly composed of polysaccharides. Genomic sequence analysis of CCMM B951 confirmed the presence of the 15-gene epsA-O operon encoding the EPS polysaccharide (contig 3, cds 110153_110905 to cds 125101_126078).

The EPS-mediated cohesive network and substrate colonisation could therefore support the presence of biofilms on apple, carrot, and orange fibres.

### Determination of antibiotic resistance properties

The presence of antibiotic-resistance genes was assessed in *B. paralicheniformis* CCMM B774, CCMM B951, CCMM B966, and CCMM B969, firstly using the CARD database^20^ and stringent sequence homologies (> 90% aa identity) with genomic sequences. Four genes were consistently identified in all strains (Table S5). The gene *ermD* encodes an rRNA methylase protein that confers resistance to erythromycin and is involved in clindamycin resistance^21^. The remaining three genes (*bcrA*, *bcrB*, and *bcrC*) are associated with resistance to bacitracin. With lower sequence homology thresholds, the genes *cat/aph* and *aadk*, known to reduce susceptibility to chloramphenicol and streptomycin in *Bacillus*, were detected in *B. paralicheniformis* with identities of no more than 57%.

The susceptibility of the selected strains to eight clinically important antibiotics was then evaluated experimentally, revealing uniform susceptibility of all strains to vancomycin, gentamycin, tetracycline, and kanamycin (with the exception of CCMM B774 for the latter). Conversely, all strains exhibited resistance to clindamicin, streptomycin, chloramphenicol, and erythromycin (Table S6), consistent with in silico prediction.

### Virulence factors and undesirable genes

To further assess the safety of *B. paralicheniformis*, predicted genes for virulence factors and toxicity were compared with those of other *Bacillus* strains such as *Bacillus anthracis* (pathogen), *B. clausii (recognised probiotic), and B. licheniformis*. Strikingly, the only toxicity gene identified for the four selected strains encoded Hemolysin III haemolytic toxin (Table S7). In addition, several other genes involved in diverse cellular functions and bacterial adaptation (including acid resistance) were detected. It is crucial to emphasize that, in the absence of confirmed pathogenic mechanisms, these genes can be regarded as beneficial, as they enhance the bacterial capacity to adapt to its environment^22^.

### Immunomodulatory properties of *B. paralicheniformis*

**Figure 8:**
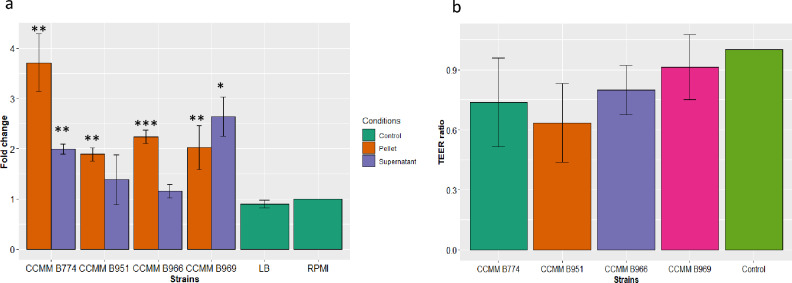
Effect of *B. paralicheniformis* CCMM strains on (**a**) NF-κB activity in HT29 cells. NF-κB activation was measured by SEAP secretion and expressed as mean ± SEM fold change toward unstimulated cells. Data represent mean ± SD (n ≥ 3). The difference from the RPMI control group was analysed by *t* test; ****P < 0.0001; ***P < 0.001; **P < 0.01; *P < 0.05; *P* > 0.05 was considered as not significant. (**b**) Barrier integrity of Caco-2 cells. TEER was measured before adding bacterial suspensions at a ratio of 40 bacteria per cell. TEER was measured a second time at the end of the 24 h of coincubation.

The capacity of *B. paralicheniformis* to activate the NF-κB transcription factor was experimentally assessed using intestinal epithelial cells (IECs) containing a reporter system, and stimulated with either bacterial lysates or extracellular medium (Fig. 8). Consistent, albeit moderate, activation of NF-κB was evidenced for all strains tested, at varying levels. Notably, LB culture medium and RPMI control showed no activity (Fig. 8a). The extracellular fluid of CCMM B969 was particularly noteworthy, exhibiting a significant 2.6-fold increase in NF-κB activation compared with the RPMI control. The CCMM B774 cell lysate showed an even higher activation ratio of approximately 4–4.5.

### Modulation of intestinal permeability in a cell model

To investigate whether *B. paralicheniformis* can maintain the integrity of the intestinal barrier, measurements of trans-epithelial electrical resistance (TEER) in Caco-2 cells were done to assess barrier integrity. Our results unveiled a strain-dependent impact on intestinal barrier integrity, but this was not significant. Interestingly, while none of the strains improved intestinal barrier integrity, *B. paralicheniformis* CCMM B969 had the TEER ratio closest to control for maintenance of barrier integrity (Fig. 8b). The other strains exhibited trends, indicating potential positive effects on barrier permeability.

## Discussion

In this study, 40 bacterial strains of the genus *Bacillus*, previously identified as *B. aerius*, were screened for their ability to degrade DFs. Among them, nine strains were able to grow on selected purified fibres (lichenan and xylan), representative of complex plant polysaccharides entering the colon, and used also apple, orange and carrot natural fibres through GHs differential expression. We therefore examined the preliminary safety properties of these strains, in accordance with EFSA recommendations for probiotic characteristics.

Molecular analyses firstly allowed the re-identification of four strains as *B. licheniformis* and five strains as *B. paralicheniformis*, as previously done for *B. paralicheniformis* CCMM B940^12^. We compared common and easy-to-use methods with more expensive and time-consuming analyses to determine a reliable, fast and cheap method for identifying strains belonging to these two closely related *Bacillus* species. Our phylogenetic analyses confirmed that the 16S rRNA genes were not discriminating enough, for both *B. licheniformis* and *B. paralicheniformis* strains^23^. Compared to MLST and genomic sequences, our results confirmed that the partial sequence of the *gyrB* housekeeping gene could be a useful and easy tool to separate these species. The *gyrB* gene sequences have been used in phylogenetic studies of the *Bacillus anthracis–cereus–thuringiensis* group^24^, as a suitable phylogenetic marker for species-level taxonomic relationships^25^. If some are not confident enough with a single gene analysis, MLST using six housekeeping genes based on the MLST schemes developed by Madslien et al.^26^ and used in this study, strongly discriminates *B paralicheniformis* from *B. licheniformis*. Our results corroborate and complement previous studies showing that the two lineages, *B. paralicheniformis* and *B. licheniformis*, are genotypically distinct^26–28^*.* Five new STs related to six *B. paralicheniformis* CCMM strains enriched the PubMLST database. The remaining *Bacillus* strains of the CCMM, including unidentified *Bacillus* spp. isolates, can be re-identified according to these validated methods.

The fibrolytic properties of *Bacillus* isolates were then examined for their capacity to use various sources of complex fibres entering the colon for food digestion, as would commensal fibrolytic gut bacteria.

Experimental assays and complementary in silico approaches have highlighted a large panel of enzymes (128 CAZYmes including 67 GHs) dedicated to the breakdown of complex polysaccharide structures corresponding to DFs, even cellulose, with a particular property of *B. paralicheniformis* for xylan degradation conferring an ecological advantage compared to *B. licheniformis*. Apple, carrot, and orange are naturally rich-fibre fruits and vegetables widely consumed in European countries, and recognised for health benefits^29^. They differ in non-digestible polysaccharides composition (according to the literature), mainly cellulose, hemicelluloses and pectin. Hemicelluloses and pectin comprise various polysaccharidic structures (in terms of sugar monomers and glycosidic bonds) that can only be degraded by a multitude of GHs with varying substrate specificities and efficiency to produce simple sugars for bacterial fermentation. Variations in plant origin and purification methods impact fibre composition and make it difficult to compare NSP contents between natural fibres. In our study, apple induced the highest extracellular xylanase activities for two of four *B. paralicheniformis* strains, including CCMM B969 (Fig. 4). This is consistent with the higher abundance of GH11 and GH30_8 xylanases in the B969 secretome for apple cultures (Fig. 6c), and could reflect the higher hemicellulose content of apple (up to 24%) compared to other substrates (less than 11%). Regarding lichenase activity, despite its lower abundance in CCMM B969 secretome from apple culture, the lichenase activity of extracellular proteins of all strains was the highest. This could indicate that other kind of GHs, such as β(1,4)-glucanases may cooperate in the degradation of apple fibres.

Growth in natural substrates could also occur first in the favor of the simple sugars readily available in fruit and vegetable peels, because CCMM strains of *B. paralicheniformis* possess monosaccharidases from GH1, GH3, and GH4 (Table S4), and glycolytic activities, targeting various monosaccharides from DFs (Table S3). Orange, apple and carrot peels are thought to contain 16.9%, 13%, and 6% of soluble sugars, respectively (Table S2).

Some studies have already reported the presence of cellulases and hemicellulases in various strains of *B. licheniformis*^30,31^. However, no information was published yet concerning *B. paralicheniformis*, except for the strain CCMM B940 of our previous study^12^. All strains studied here had higher specific activities than our reference strain CCMM B940^12^, suggesting that *B. paralicheniformis* species bears a significant function in fibre degradation. This finding was confirmed by the genomic features of these strains, which revealed a complete set of GH genes dedicated to the degradation of various polysaccharides from DFs, such as xylans, mixed-linkage polysaccharides, pectin, mannans, fructans, starch, and even cellulose that no commensal human gut bacteria are so far able to degrade. According to the CAZy database classification^4^, nearly 67 GH genes and 8 PL genes dedicated to plant cell wall carbohydrate degradation were evidenced per *B. paralicheniformis* strain, representing 32 GH families and 5 different PL families, respectively. The presence of different genes encoding CAZymes was previously predicted from the genome sequence of *B. paralicheniformis* MDJK30, isolated from soil^32^, but with 2 times fewer GH genes and no experimental validation. To date, 14 sequences of *B. paralicheniformis* genomes annotated for CAZymes genes are listed in the CAZy database, all from unpublished works (https://www.cazy.org, updated 2022/10/18).

Our studies using CCMM strains combined in silico genomics and in vitro experiments to demonstrate the efficiency of *B. paralicheniformis* in degrading complex DFs. Genomics can provide information on the functional potential of microorganisms, but it does not imply that the predicted phenotype is expressed. Here, we confirmed the presence of CAZymes in the secretome of *B. paralicheniformis* CCMM B969, the most active strain, by mass spectrophotometry. The expression of different GHs in the secretome, particularly xylanases, lichenases, and cellulases involved in DF degradation, supports the synergistic activity of various glycosidases required for the breakdown of complex DFs to fermentable monosaccharides^33^. Interestingly, the enzyme abundance was regulated by carbon sources, as often observed for bacterial glycoside hydrolases^34–36^. This is the first time the fibrolytic function of *B. paralicheniformis* has been evidenced at both the genomic and secretomic levels, with enzyme production regulated according to the fibre present in the culture medium. This is also the first time EPS has been observed for *B. paralicheniformis* using SEM, supported by genomic analysis. Previous works have evidenced EPS in *Bacillus* species, including its closest phylogenetic relative, *B. licheniformis*^37^. As mentioned above, one should be careful with studies including identification of *Bacillus* isolates based solely on rDNA sequencing, as it is not enough discriminant to distinguish between the two species. EPS from probiotic bacteria are Generally Regarded As Safe (GRAS). Depending of their chemical structure (sugar composition, glycosidic linkages), they may confer beneficial properties such antimicrobial, anti-tumor, anti-inflammatory, anti-diabetic, cholesterol lowering and immunomodulatory activities^37–39^. Microbial EPS are, thus, of increasing interest for health-promoting effects as they could be natural substitutes for synthetic drugs in therapeutic applications. It is therefore particularly interesting to find EPS in *B. paralicheniformis* as a promising beneficial microbe.

Using different fruits and vegetables as unique carbon sources, we showed that 4 strains of *B. paralicheniformis*, especially the strain CCMM B969, can produce different GHs specialized in complex DFs breakdown. These fruits and vegetables are essential foods in the daily diet and are rich in DFs^33,40^. It is well established now that DFs provide substrates to gut microbiota for the production of different metabolites that could significantly impact the host’s health. Species belonging to Firmicutes and Actinobacteria are the main responders to DFs, although they contain relatively few fibre-metabolizing enzymes per organism^2,41^. However, they generally have more specialized roles, such as initiating complex substrate degradation^2^. These species are considered “specialists” in contrast with “generalists” such as *Bacteroides* species that encode between 100 to over 300 CAZymes^42^. Primary degraders play a crucial role in the cross-feeding network, and they are the first bacteria to colonize and initiate the degradation of complex carbohydrates^43^. The present strain CCMM B969 contains more GHs and PLs than the mean (74 compared to 39.6) per genome in the Firmicutes phylum^44^. Likewise, it contains more CAZymes than the *Butyrivibrio fibrisolvens* 16/4 and *Ruminococcus champanellensis* 18P13 (128 compared to 115, and 87, respectively) isolated from the human intestine^35^. The CCMM B969 strain could be considered a "specialist" as it secretes specific GHs such as GH11, GH16, and GH30_8 which can be effective in initiating the deconstruction of complex DFs. This thus provides ecological relevance to CCMM B969 in light of applications as beneficial microbes, as it could act as a pioneer degrader of complex DFs in the gut, and release beneficial oligosaccharides and metabolites to secondary degraders, thereby improving SCFAs production for bacteria and for the host. By supporting microbial trophic networks, B969 could act as health-promoting beneficial microbes, especially in the context of metabolic syndrome.

To assess the safety of *B. paralicheniformis* CCMM strains, in particular CCMM B969, we conducted a comprehensive study aligned with EFSA recommendations for probiotic candidates. Various *Bacillus* species have been evaluated by EFSA, and received the "Qualified Presumption of Safety" (QPS) status, confirming their safety for human consumption^45^. These strains meet specific criteria: (1) strain and species identification, (2) absence of transferable antimicrobial resistance, and (3) absence of toxinogenic activity^45–47^. The combined in vitro and in silico analysis of *B. paralicheniformis* CCMM strains for antibiotic resistance genes and susceptibility profiles, revealed resistance to erythromycin, clindamycin, streptomycin, and chloramphenicol but was sensitive to gentamicin, vancomycin, tetracycline, and kanamycin. These resistances have previously been reported in *B. licheniformis* and *B. paralicheniformis*^21,48,49^, suggesting that the antibiotic resistances observed in these species could be intrinsic and not associated with mobile genetic elements^49^. Thus, fibrolytic strains of *B. paralicheniformis*, such as the CCMM B969 candidate as beneficial microbes, could therefore exhibit natural resistance to clindamycin, erythromycin, streptomycin, and chloramphenicol, as some current *Bacillus* probiotics probably do. In addition, virulence and toxicity assessment of the CCMM isolates evidenced the predicted hemolysin III gene which has already been identified in marketed probiotics recognized as safe, such as *Lactiplantibacillus plantarum* and *Bacillus clausii* strains^22^. Strains of *Bacillus subtilis* and *B. amyloquefaciens* also carry this gene, but have no hemolytic activity^50,51^. These results suggest the potential absence of toxic activity for the beneficial microbe candidate CCMM B969.

IECs represent the first line of contact with the intestinal microbiota and play a fundamental role in regulating intestinal immune processes related to various pathologies (cancer, chronic inflammatory diseases, metabolic diseases).* B paralicheniformis* CCMM B969 exhibited moderate to low activation of the NF-κB transcription factor in IECs, indicating a balanced immunostimulatory profile with activation levels comparable to those found in the human commensal *Akkermansia muciniphila*^52^. *A. muciniphila* has a positive impact on host health, particularly by promoting intestinal barrier integrity^53,54^. This preliminary result paints a first picture of the interaction of our beneficial microbe candidate with IECs, showing no excessive activation or inhibition of NF-κB. The pro- or anti-inflammatory profile of CCMM B969 will nevertheless require further investigation, including cytokine production assays.

In summary, the genomic and phenotypic characteristics of *B. paralicheniformis* CCMM B969 confirm that the strain has no significant safety issues, such as toxicity for human consumption or transfer of antibiotic resistance to the gut microbiota. In light of these results and the long history of safe consumption of *B. paralicheniformis* in traditional fermented soy-based foods, *B. paralicheniformis* CCMM B969 is a promising candidate as beneficial microbes in humans. Preclinical models in the context of metabolic syndrome could be useful to confirm the benefits for the gut microbiota.

Exploring the fibrolytic potential, safety, and immunomodulatory properties of *B. paralicheniformis* has opened new perspectives on the role *B. paralicheniformis* in improving the main function of the gut microbial ecosystem, and significantly impacting intestinal health and overall well-being.

## Methods

### Bacterial strains, fibres, and culture conditions

*Bacillus* strains, previously identified as *B. aerius* from Moroccan extremophilic ecosystems (desert, hot springs, salt marshes), were obtained from the CCMM (https://www.ccmm.ma)^16^. Strains were selected regarding the history of probiotics strains from the genus *Bacillus* for health-promoting applications, spore-producing ability conferring resistance in the gastro-intestinal tract, the polysaccharidolytic properties (cellulase, amylase) of the CCMM strains reported previously (Aanniz et al.^14^), their capacity to grown on mesophilic conditions, and their phylogenetic proximity with strains of *Bacillus licheniformis* as this species has current probiotics applications/properties. Strains, their origin and growth temperature are listed in Table S1. Forty (40) strains cryopreserved at -80 °C were aerobically cultivated twice on Luria–Bertani (LB) agar medium (BD Difco, Sparks, Maryland, USA) for 18 h at 37 °C. Individual colonies were then picked and grown at 37 °C in BSS agar or broth supplemented with purified or natural fibres (0.5%) as the sole carbon source, as described in Maski et al.^12^. The BSS medium (w/v) from Parab et al.^55^ was modified as follows: NaCl, 6.0 g; MgSO_4_, 7.0 g; NH_4_Cl, 1.0 g; K_2_HPO_4_ (10%), 35 mL; KH_2_PO_4_ (10%), 10 mL; trace metal solution mix A5 with Co (Sigma-Aldrich, Saint-Quentin-Fallavier, France) 1.0 mL.

### Natural fibre sources and Polysaccharides

For pure bacterial cultures, Icelandic moss lichenan, Tamarind xyloglucan, and beechwood xylan (Megazyme, Wicklow, Ireland) were chosen as representative polysaccharides of hemicelluloses, the main constituents of plant cell walls. Natural fibres were prepared from certified organic fruits and vegetables. Briefly, the orange (flavedo and albedo), apple, and carrot were peeled and dried for at least 24 h at 60 °C and crumbled finely using an electric grinder. The dried peels were crushed and then sieved to obtain a homogeneous powder.

The chromogenic AZCL-Xyloglucan (Tamarind) and AZCL-Xylan (Beechwood) polysaccharides from Megazyme (Wicklow, Ireland) were used for enzyme assays.

### Functional screening of the *Bacillus* sp. CCMM strains

Functional screening for xylanase and lichenase activities was performed as follows: after growth in LB broth, bacteria were striated on the surface of Petri dishes containing two layers of medium: a first one of 20 mL BSS agar medium without any antibiotics and a second one, spread out in overlay, of 15 mL BSS agar medium supplemented with 0.5% (wt/v) of purified polysaccharide.

Cells grew into colonies at 37 °C for 2 days, and then colonies were softly removed from the agar surface. The plates were then flooded with 10 mL of 0.1% Congo red (Sigma Aldrich, Saint-Quentin-Fallavier, France) for 30 min. and washed off three times with 10 mL of 1 M NaCl for 15 min. Clear zones appeared around polysaccharidolytic colonies.

### Protein samples and enzyme assays

To prepare protein samples, each strain was cultivated twice at 37 °C for 48 h in BSS medium supplemented with natural fibres of apple, carrot, or orange as described above. The cultures (30 mL) were then centrifuged at 5000*g* for 15 min at 4 °C. The supernatants were filtered through a 0.22 μm Millipore filter and concentrated 25-fold with a Corning Spin-X UF 10 K MWCO concentrator at 4 °C following the manufacturer’s protocol (Corning B.V., Amsterdam, The Netherlands). Samples were stored at − 20 °C before use.

Agar-well plate enzymatic assays were performed to detect xylanase, lichenase, and xyloglucanase activities of 25-fold concentrated extracellular proteins. Samples were loaded (60 μL/well) in Petri dishes containing 35 mL of BSS medium supplemented with 0.1% chromogenic AZCL-Xyloglucan, AZCL-Xylan, or 0.1% non-chromogenic lichenan (all from Megazyme, Wicklow, Ireland). The plates were incubated overnight at 37 °C, and those with non-chromogenic polysaccharides were stained with Congo red as described for the functional screening. Clear or blue halos revealed substrate degradation around wells containing polysaccharidolytic enzymes.

Protein concentration was determined by the Bradford method^56^, with bovine serum albumin as the standard. Extracellular hemicellulolytic activities of the strains were quantified using the dinitrosalisylic acid (DNS) colorimetric method to determine reducing sugars as described by Miller^57^. The number of enzymes and the incubation time were chosen to provide assay conditions in which the measured activity was proportional to these two parameters. Reducing sugars released after enzyme–substrate incubations were quantified in triplicate using glucose or xylose as standard. One international unit (IU) was defined as the amount of enzyme which produced 1 µmol of reducing sugars in 1 min.

The API50CHB system (BioMérieux, Craponne, France), including strips for 49 miniature enzyme assays and inoculation medium, was used to determine the carbohydrate fermentation pattern of *Bacillus* strains grown on LB agar plates at 37 °C for 24h. Selected colonies of the pure culture were suspended in API50CHB medium according to the manufacturer’s recommendations, and the resulting suspension was used for inoculation of the carbohydrate-containing wells. The strips were incubated at 37 °C for 48h according to the manufacturer’s instructions. The experiment was performed twice per strain.

### Scanning electron microscopy

The strain *B. paralicheniformis* CCMM B951 was cultured for 48 h at 37 °C in BSS with various natural fibres as described above. Samples immersed in a fixative solution (2.5% glutaraldehyde in 0.2 M sodium cacodylate buffer, pH 7.4) were deposited on sterile cover-glasses discs (Marienfeld, VWR, France) and stored for 1 h at room temperature and overnight at 4 °C. The fixative was removed, and samples were rinsed three times for 10 min in the sodium cacodylate solution (pH 7.4). The samples underwent progressive dehydration by soaking in a graded series of ethanol (50–100%) before critical-point drying under CO_2_. Samples were mounted on aluminum stubs (15 mm diameter) with carbon adhesive discs (Agar Scientific, Oxford Instruments SAS, Gometz-la-Ville, France) and sputter coated with platinum (Polaron SC7640, Milexia, Verrières-le-Buisson, France) for 220 s at 10 mA. Samples were visualized by field emission gun scanning electron microscopy (SEM FEG). They were viewed as secondary electron images (2 kV) with a Hitachi SU5000 instrument (Milexia, Verrières-le-Buisson, France). Samples preparation and scanning Electron Microscopy analyses were performed at the Microscopy and Imaging Platform MIMA2 (MIMA2, INRAE, 2018. Microscopy and Imaging Facility for Microbes, Animals and Foods, https://doi.org/10.15454/1.5572348210007727E12).

### Preparation and analysis of *B. paralicheniformis* CCMM B969 secretome

The secretome was prepared from the 25-fold concentrated extracellular proteins of *B. paralicheniformis* CCMM B969 cultures, as described in protein samples and enzyme assays section. The concentrated secretome was purified after short migration on SDS–polyacrylamide gel electrophoresis (SDS-PAGE). After the excision of gel bands, proteins were identified at PAPPSO platform facilities (http://pappso.inra.fr/). Briefly, gel bands were washed with 10 mM dithiothreitol (Sigma-Aldrich, Saint-Quentin-Fallavier, France) and 55 mM iodoacetamide (Sigma-Aldrich). In-gel tryptic digestion was performed overnight with 200 ng of trypsine (Promega, Charbonnières-les-Bains, France) in 50 mM bicarbonate (Sigma-Aldrich) buffer at 37 °C. Peptides were extracted with 0.5% trifluoroacetic acid (TFA) in 50% acetonitrile (ACN), dried and re-suspended in 60 µL of 0.1% TFA in 2% ACN for analysis in high-resolution mass spectrometry.

Four µL of each sample were injected into an Ultimate 3000 RSLC system (Thermo Fisher Scientific) coupled to an Orbitrap Fusion™ Lumos™ Tribrid™ (Thermo Fisher Scientific) by nanoelectrospray ion source. Protein digest was injected and preconcentrated on a precolumn (Acclaim PepMap C18 particle 5 µm size, 5 mm length, 300 µm i.d., Thermo Fisher Scientific) at 20 µL/min with 0.08% TFA in 2% ACN in 2 min, followed by a separation on reverse phase separating column (Acclaim PepMap RSLC nanoViper, C18 particle 3 µm size, 500 mm length, 75 µm i.d., Thermo Fisher Scientific). Buffers were 0.1% formic acid in 98% water (solvent A) and 0.1% formic acid in 80% ACN (solvent B). The peptides were eluted with a multi-step gradient from 1 to 45% of solvent B for 55 min at 300 nL/min for a total run of 65 min. MS data acquisition included a full MS scan covering 300–1600 mass-to-charge ratio (*m/z*) with a resolution of 120,000. HCD fragmentation (MS/MS) step was performed with a normalised collision energy of 30 and a resolution of 30,000.

All MS/MS spectra were searched against *Bacillus (para)licheniformis* database using X!TandemPipeline version 0.4.19^58^, the open search engine developed by PAPPSO (http://pappso.inra.fr/bioinfo/xtandempipeline/). Precursor and fragment mass tolerance was 10 ppm. Data filtering was achieved according to a peptide E-value < 0.01, protein E-value < 10e-4 and to a minimum of two identified peptides per protein. Contaminant peptides were discarded following identification using a standard proteomics contaminant database. MassChroQ software (v. 2.2.27) was employed to perform alignment and ion chromatograms extraction (XIC) defined as the sum of the MS1 intensities of all peptides associated with a protein for quantification^59^.

Relative quantification of the proteins was calculated using R scripts by analyzing peptide intensities obtained from XIC. Firstly, reliable peptides were selected according to the criteria described in Belouah et al.^60^, i.e., shared peptides, uncorrelated peptides, peptides showing retention time instability, and peptides showing missing data were filtered out. Protein relative abundances were computed as the sum of the peptide intensities. Proteins showing a p value corrected for multiple comparisons < 0.05 were considered as showing a significant variation.

### DNA isolation, PCR amplification, and sequencing of 16S rRNA and gyrB genes

Genomic DNA was isolated from *Bacillus* cells harvested as described above, using Wizard® Genomic DNA Purification Kit (Promega, Charbonnières-les-Bains, France) according to the supplier’s protocol. PCR amplification of the complete 16S rRNA gene (approx. 1500 bp) was performed using the FD1 (5′-AGAGTTTGATCCTGGCTCAG-3′) and RP2 (5′-ACGGCTACCTTGTTACGACTT-3′) universal primers^61^. PCRs were carried out with genomic DNA (150 ng) and 1X Phusion Green Hot Start II High-Fidelity PCR Master Mix (Thermo Fisher Scientific, Illkirch, France) following the supplier’s instruction with the annealing temperature of 55 °C (25 cycles).

The amplified 16S rRNA encoding genes were sequenced full length in both directions using Sanger-technology (Eurofins Genomics, Cologne, Germany).

The taxonomic assignment of complete 16S rRNA encoding gene sequences was based on nucleic sequence similarity using the microbial nucleotide Basic Local Alignment and Search Tool (Blast) at NCBI facilities with either representative genomes, complete genomes, or the non-redundant nucleotide database nt (identity percentage > 97%). The MEGA X software was used for phylogenetic analysis and tree construction using the Neighbor-Joining method^62,63^.

The partial sequence of the gene encoding the subunit B protein of DNA gyrase (*gyrB*) was included in the phylogenetic analysis for comparative purposes. The *gyrB* fragments of approximately 400 bp were amplified from *Bacillus* genomic DNA using specific PCR primers^26^, and sequenced at Eurofins (Cologne, Germany).

### MLST analysis

Intragenic regions of six housekeeping genes of the *Bacillus* genus (*adk, ccpA, recF, rpoB, spo0A, sucC)* were selected for MLST analysis^26^. All primers were synthesized by Eurofins (Constance, Germany). PCR amplifications were performed using Phusion Green Hot Start II high fidelity PCR Master Mix (Thermo Fisher Scientific, Illkirch, France) as described previously. Purified PCR products were sequenced by Eurofins (Cologne, Germany). The sequences were aligned and concatenated with MEGA X software. The PubMLST database was used to construct the MLST phylogenic tree^64^ using the *B. licheniformis* database.

### Genomic analyses

Genomic DNAs were sequenced at Eurofins Genomics (Constance, Germany) using Illumina HiSeq technology for whole genome sequencing. The libraries were prepared with 2 × 150-bp paired-end read length, including DNA fragmentation, adapter ligation, amplification, and size selection. All the steps were performed according to Eurofins Genomics protocols, producing between 5 and 7 million reads depending on the strain. Trimming, assembly, and quality assessment of the genome sequences were done as described previously^12^. The genomes were then annotated using the default parameters of Prokka 1.14^65^.

Assignment of ORFs to CAZy families was done as for the daily updates of the CAZy database^4^. Functional annotations were performed according to Blastp hits against sequences of biochemically characterized enzymes listed in the CAZy database^4^ on January 4, 2023. Function assignments were made using the function of the best hit with at least 50% identity and > 95% coverage.

### Safety assessment of selected strains as potential probiotics

#### Determination of virulence factors and undesirable genes

The safety of the selected strains was assessed by confirming the absence of virulence factors in their genomes. Genomic data were compared with sequences of known virulence factors or proteins involved in their synthesis, curated from the Virulence Factor Database (VFDB).

#### Determination of antibiotic resistance properties

Antibiotic resistance genes within the genomes of the selected strains were identified using publicly available databases, specifically the Comprehensive Antibiotic Resistance Database (RGI 6.0.2, CARD 3.2.7), accessible at https://card.mcmaster.ca/analyze/rgi using the stringent default settings.

Phenotypic antibiotic susceptibility was determined against nine antibiotics recommended by EFSA, including ampicillin, vancomycin, gentamicin, kanamycin, streptomycin, erythromycin, clindamycin, tetracycline, and chloramphenicol (Sigma-Aldrich Co., St. Louis, MO, USA). Bacterial suspensions of the selected probiotic candidates (10^6^ CFU/mL) and each antibiotic (ranging from 512 to 1 mg/L) were mixed in 96-well microplates and incubated at 37 °C for 18 h. The optical density of the mixtures was measured using a microplate reader (Infinite 200, Tecan), and the minimum inhibitory concentrations (MICs) were determined as the lowest concentrations that completely inhibited bacterial growth. Antibiotic susceptibility was assessed based on cutoff values established in accordance with EFSA guidelines^46^.

### Cell stimulation and reporter systems assays

Cell culture, stimulation, and reporter system assays were performed following the procedures outlined by Martin-Gallausiaux et al.^52^. HT29 cells (American Type Culture Collection, ATCC) were cultured in RPMI 1640 GlutaMAX™ medium supplemented with 10% heat-inactivated fetal bovine serum (FBS, Eurobio), 50 IU/mL penicillin, 50 μg/mL streptomycin, 10% (v/v) 100 mM Hepes, and 10 mM non-essential amino acids. We utilized HT29 cells stably expressing the secreted alkaline phosphatase (SEAP) reporter gene under the control of the NF-κB pathway (pNifty, Invivogen) for monitoring NF-κB activation.

HT29-NF-κB cells were seeded at a density of 3.10^4^ cells per well in 96-well plates, 24 h prior to incubation with bacterial supernatants, pellets, or reagents. Following a 24-h stimulation with bacterial supernatants, pellets, or controls (RPMI and non-inoculated bacterial culture medium LB) in a total culture volume of 100 µL per well, the reporter assay was performed. SEAP activity was quantified using the Quanti-Blue reagent (Invivogen) and measured with a microplate reader (655 nm, Infinite 200, Tecan). NF-κB activation levels were normalized to unstimulated cells. Experiments were conducted in triplicates for a minimum of three independent assays.

### Study of intestinal permeability by measurement of transepithelial electrical resistance (TEER)

TEER measurements were performed according to the procedure of Chamignon et al.^66^. Briefly, Caco-2 cells were cultured in Dulbecco’s modified Eagle’s medium (DMEM) supplemented with 20% heat-inactivated fetal bovine serum, 1% nonessential amino acids, and 1% streptomycin/penicillin on Transwell® 24-well plate (Transwell, 0.33 cm^2^, 0.4 μm, Corning) until they reached a TEER value of > 900 Ω.cm^−2^. The impact of bacteria on various epithelial barriers was evaluated by measuring TEER in Caco-2 cells. TEER values were recorded before bacterial addition (t = 0 h) and after 24 h of incubation. Bacterial suspensions were added at a ratio of 40 bacteria per cell. DMEM medium was used as a negative control. The TEER ratio was calculated as follows, and the bacterial effects were assessed by comparison with the controls.

$${\text{TEER ratio }} = \, \left( {{\text{TEER at t24 }} / {\text{ TEER at t}}0} \right) \, / \, \left( {{\text{Control TEER at t24 }} / {\text{ Control TEER at t}}0} \right)$$

### Supplementary Information

Supplementary Information 1.

Supplementary Information 2.

Supplementary Information 3.

Supplementary Information 4.

Supplementary Information 5.

Supplementary Information 6.

Supplementary Information 7.

Supplementary Information 8.

## Data Availability

The assembled genomes generated during this study have been deposited in the European Nucleotide Archive (ENA) at EMBL-EBI under accession number PRJEB58735 (https://www.ebi.ac.uk/ena/browser/view/PRJEB58735). The associated BioSamples are SAMEA112306458, SAMEA112306457, SAMEA112306425, and SAMEA112306424 for *B. paralicheniformis* CCMM B943, CCMM B774, CCMM B969, and CCMM B951, respectively. For the strain CCMM B940, see Project PRJNA680612 by Maski et al.^12^.
