# Supplementary material for: Exploring the hemicellulolytic properties and safety of Bacillus paralicheniformis as stepping stone in the use of new fibrolytic beneficial microbes
Source: Sci Rep. 2023 Dec 20;13:22785. doi: 10.1038/s41598-023-49724-8 (PMC10740013; doi:10.1038/s41598-023-49724-8)

**Figure S1.** Neighbor-joining phylogenetic tree based on the housekeeping gene *gyrB* sequences of *Bacillus* isolates.
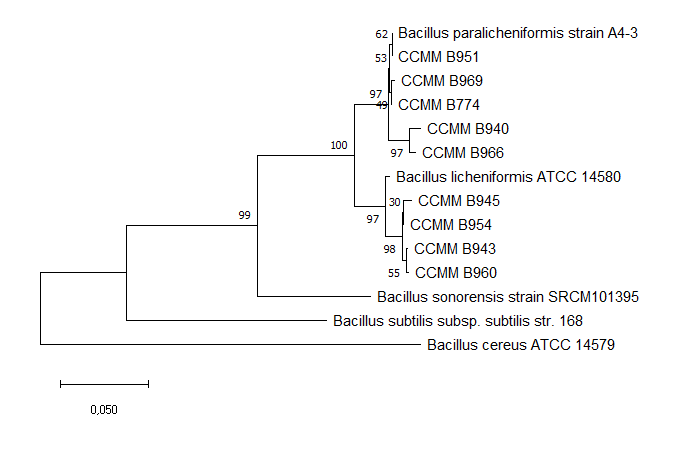

Supplement: Supplementary file 1 — Supplementary Information 1. [file 41598_2023_49724_MOESM1_ESM.docx]
