# Supplementary material for: Exploring the hemicellulolytic properties and safety of Bacillus paralicheniformis as stepping stone in the use of new fibrolytic beneficial microbes
Source: Sci Rep. 2023 Dec 20;13:22785. doi: 10.1038/s41598-023-49724-8 (PMC10740013; doi:10.1038/s41598-023-49724-8)
Supplement: Supplementary file 2 — Supplementary Information 2. [file 41598_2023_49724_MOESM2_ESM.docx]

**Table S1.** Description of *Bacillus aerius* strains from CCMM, sample sites, growth temperature, re-identified species name and MLST sequence types.

|  |  |  |  |  |  | MLST allelic type^3^ | | | | | |  |
| --- | --- | --- | --- | --- | --- | --- | --- | --- | --- | --- | --- | --- |
| *Bacillus* CCMM strain | Species (previous name^1^) | Re-identified species^2^ | Original sample site | Moroccan region | Growth temp (°C) | adk | ccpA | recF | rpoB | spo0A | susC | Sequence type (ST) |
| B770 | *Bacillus aerius* | ND | desert sand | Merzouga | 30-55 |  |  |  |  |  |  |  |
| B773 | *Bacillus aerius* | ND | desert sand | Merzouga | 30-55 |  |  |  |  |  |  |  |
| B774 | *Bacillus aerius* | *Bacillus paralicheniformis* | desert sand | Merzouga | 30-55 | 3 | 2 | 5 | 3 | 17 | 13 | 55 (new) |
| B776 | *Bacillus aerius* | ND | desert sand | Merzouga | 30-55 |  |  |  |  |  |  |  |
| B780 | *Bacillus aerius* | ND | desert sand | Merzouga | 30-55 |  |  |  |  |  |  |  |
| B792 | *Bacillus aerius* | ND | salt marsh | Sidi Abed El-Jadida | 30-55 |  |  |  |  |  |  |  |
| B821 | *Bacillus aerius* | ND | desert sand | Merzouga | 30-55 |  |  |  |  |  |  |  |
| B837 | *Bacillus aerius* | ND | desert sand | Merzouga | 30-55 |  |  |  |  |  |  |  |
| B844 | *Bacillus aerius* | ND | desert sand | Merzouga | 30-55 |  |  |  |  |  |  |  |
| B937 | *Bacillus aerius* | ND | hot spring | Ain Allah Fez | 30-55 |  |  |  |  |  |  |  |
| B938 | *Bacillus aerius* | ND | hot spring | Ain Allah Fez | 30-55 |  |  |  |  |  |  |  |
| B939 | *Bacillus aerius* | ND | hot spring | Ain Allah Fez | 30-55 |  |  |  |  |  |  |  |
| B940 | *Bacillus aerius* | *Bacillus paralicheniformis* | hot spring | Ain Allah Fez | 30-55 | 3 | 17 | 5 | 3 | 12 | 3 | 56 (new) |
| B941 | *Bacillus aerius* | ND | desert sand | Merzouga | 30-55 |  |  |  |  |  |  |  |
| B942 | *Bacillus aerius* | ND | desert sand | Merzouga | 30-55 |  |  |  |  |  |  |  |
| B943 | *Bacillus aerius* | *Bacillus licheniformis* | desert sand | Merzouga | 30-55 | 2 | 9 | 1 | 2 | 1 | 1 | 13 |
| B945 | *Bacillus aerius* | *Bacillus licheniformis* | desert sand | Merzouga | 30-55 |  |  |  |  |  |  |  |
| B946 | *Bacillus aerius* | ND | desert sand | Merzouga | 30-55 |  |  |  |  |  |  |  |
| B947 | *Bacillus aerius* | ND | desert sand | Merzouga | 30-55 |  |  |  |  |  |  |  |
| B949 | *Bacillus aerius* | ND | hot spring | Ain Allah Fez | 30-55 |  |  |  |  |  |  |  |
| B950 | *Bacillus aerius* | ND | hot spring | Ain Allah Fez | 30-55 |  |  |  |  |  |  |  |
| B951 | *Bacillus aerius* | *Bacillus paralicheniformis* | hot spring | Abaynou Guelmim | 30-55 | 3 | 18 | 5 | 3 | 2 | 3 | 57 (new) |
| B952 | *Bacillus aerius* | ND | hot spring | Abaynou Guelmim | 30-55 |  |  |  |  |  |  |  |
| B953 | *Bacillus aerius* | ND | hot spring | Ain Jerry Meknes | 30-55 |  |  |  |  |  |  |  |
| B954 | *Bacillus aerius* | *Bacillus licheniformis* | salt marsh | Sidi Moussa El-Jadida | 30-55 |  |  |  |  |  |  |  |
| B955 | *Bacillus aerius* | ND | hot spring | Abaynou Guelmim | 30-55 |  |  |  |  |  |  |  |
| B956 | *Bacillus aerius* | ND | salt marsh | Sidi Abed El-Jadida | 30-55 |  |  |  |  |  |  |  |
| B957 | *Bacillus aerius* | ND | salt marsh | Sidi Moussa El-Jadida | 30-55 |  |  |  |  |  |  |  |
| B958 | *Bacillus aerius* | ND | salt marsh | Sidi Moussa El-Jadida | 30-55 |  |  |  |  |  |  |  |
| B959 | *Bacillus aerius* | ND | salt marsh | Sidi Abed El-Jadida | 30-55 |  |  |  |  |  |  |  |
| B960 | *Bacillus aerius* | *Bacillus licheniformis* | salt marsh | Sidi Abed El-Jadida | 30-55 | 6 | 1 | 1 | 2 | 1 | 2 | 58 (new) |
| B961 | *Bacillus aerius* | ND | salt marsh | Sidi Moussa El-Jadida | 30-55 |  |  |  |  |  |  |  |
| B962 | *Bacillus aerius* | ND | salt marsh | Sidi Abed El-Jadida | 30-55 |  |  |  |  |  |  |  |
| B963 | *Bacillus aerius* | ND | desert sand | Merzouga | 30-55 |  |  |  |  |  |  |  |
| B964 | *Bacillus aerius* | ND | desert sand | Merzouga | 30-55 |  |  |  |  |  |  |  |
| B965 | *Bacillus aerius* | ND | desert sand | Merzouga | 30-55 |  |  |  |  |  |  |  |
| B966 | *Bacillus aerius* | *Bacillus paralicheniformis* | desert sand | Merzouga | 30-55 | 3 | 2 | 5 | 3 | 17 | 13 | 55 (new) |
| B967 | *Bacillus aerius* | ND | desert sand | Merzouga | 30-55 |  |  |  |  |  |  |  |
| B968 | *Bacillus aerius* | ND | desert sand | Merzouga | 30-55 |  |  |  |  |  |  |  |
| B969 | *Bacillus aerius* | *Bacillus paralicheniformis* | desert sand | Merzouga | 30-55 | 3 | 10 | 3 | 3 | 7 | 3 | 54 (new) |

modified from Aanniz et al.^16^, and the CCMM website (www.ccmm.ma)

^1^ according to Aanniz et al.^16^

^2^ this study (except for B940, from Maski et al.^12^)

^3^this study. New sequence types (STs) generated from the PubMLST datatbase with *Bacillus* CCMM strains have been marked "new".

ND: Not determined

**NGOM SI *et al.*** Exploring the hemicellulolytic properties and safety of *Bacillus paralicheniformis* as stepping stone in the use of new fibrolytic beneficial microbes (Scientific Reports)
