# Supplementary material for: Exploring the hemicellulolytic properties and safety of Bacillus paralicheniformis as stepping stone in the use of new fibrolytic beneficial microbes
Source: Sci Rep. 2023 Dec 20;13:22785. doi: 10.1038/s41598-023-49724-8 (PMC10740013; doi:10.1038/s41598-023-49724-8)
Supplement: Supplementary file 3 — Supplementary Information 3. [file 41598_2023_49724_MOESM3_ESM.docx]

**Table S2** Insoluble Fibers and soluble sugars content in orange, apple, and carrot peels.

|  | **Composition (%)** | | |
| --- | --- | --- | --- |
| **Substrate** | **Orange^1^** | **Apple^2^** | **Carrot^3^** |
| Fraction |  |  |  |
| Lignin | 0.8 | 15.3-23.5 | 2.5 |
| Cellulose | 9.2 | 7.2-43.6 | 80.9 |
| Hemicellulose | 10.5 | 4.3-24.4 | 9.1 |
| Pectin | 42.5 | 3.5-14.3 | 7.4 |
| Soluble sugars | 16.9 | < 13.0 | < 5.5 |

^1^ according to Rivas et al., (2008), doi 10.1021/jf073388r

^2^ according to Dhillon et al., (2013), doi 10.1016/j.rser.2013.06.046

^3^ according to Conversa et al., (2021), doi 10.3390/foods11010045, and Sharma et al., (2012), doi 10.1007/s13197-011-0310-7

**NGOM SI *et al.*** Exploring the hemicellulolytic properties and safety of *Bacillus paralicheniformis* as stepping stone in the use of new fibrolytic beneficial microbes (Scientific Reports)
