# Supplementary material for: Exploring the hemicellulolytic properties and safety of Bacillus paralicheniformis as stepping stone in the use of new fibrolytic beneficial microbes
Source: Sci Rep. 2023 Dec 20;13:22785. doi: 10.1038/s41598-023-49724-8 (PMC10740013; doi:10.1038/s41598-023-49724-8)
Supplement: Supplementary file 4 — Supplementary Information 4. [file 41598_2023_49724_MOESM4_ESM.docx]

**Table S3.** Carbohydrate fermentation patterns of four *B. paralicheniformis* CCMM strains using API50CHB.


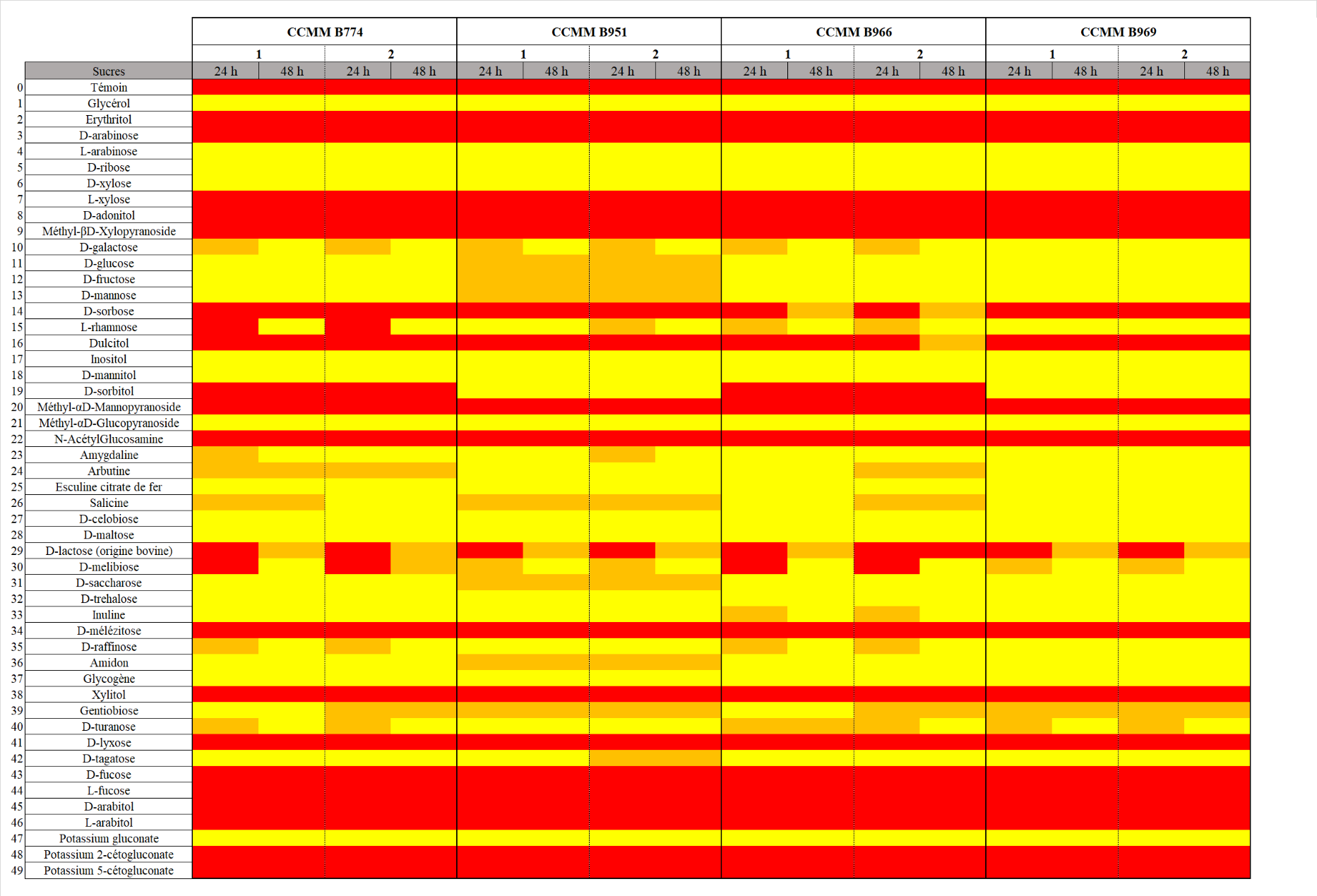


Red: negative; orange: intermediary; yellow: positive.

**NGOM SI *et al.*** Exploring the hemicellulolytic properties and safety of *Bacillus paralicheniformis* as stepping stone in the use of new fibrolytic beneficial microbes (Scientific Reports).
