# Supplementary material for: Exploring the hemicellulolytic properties and safety of Bacillus paralicheniformis as stepping stone in the use of new fibrolytic beneficial microbes
Source: Sci Rep. 2023 Dec 20;13:22785. doi: 10.1038/s41598-023-49724-8 (PMC10740013; doi:10.1038/s41598-023-49724-8)
Supplement: Supplementary file 7 — Supplementary Information 7. [file 41598_2023_49724_MOESM7_ESM.docx]

**Table S6** Antimicrobial resistance phenotype of selected *Bacillus paralicheniformis* strains

|  | Minimal Inhibitory concentration (MIC) (mg/L) | | | | | | | | |
| --- | --- | --- | --- | --- | --- | --- | --- | --- | --- |
| Strains | Kanamycin | Clindamicin | Streptomycin | Chloramphenicol | Vancomycin | Erythromycin | Gentamycin | Tetracyclin |  |
| CCMM B774 | 32 | 512 | 32 | 64 | 1 | 512 | 1 | 1 |  |
| CCMM B951 | 8 | 512 | 512 | 64 | 2 | 512 | 4 | 8 |  |
| CCMM B966 | 8 | 512 | 128 | 128 | 2 | 512 | 1 | 4 |  |
| CCMM B969 | 8 | 8 | 512 | 32 | 1 | 512 | 1 | 8 |  |
| EFSA cut-off | 8 | 4 | 8 | 8 | 4 | 4 | 4 | 8 |  |

**NGOM SI *et al.*** Exploring the hemicellulolytic properties and safety of *Bacillus paralicheniformis* as stepping stone in the use of new fibrolytic beneficial microbes (Scientific Reports).
